# Hypomethylation in promoters of PGC-1α involved in exercise-driven skeletal muscular alterations in old age

**DOI:** 10.1515/biol-2022-0959

**Published:** 2024-09-10

**Authors:** Qiaowei Li, Qin Liu, Zhong Lin, Wenwen Lin, Feng Huang, Pengli Zhu

**Affiliations:** Shengli Clinical Medical College of Fujian Medical University, Fuzhou, 350001, P. R. China; Fujian Provincial Institute of Clinical Geriatrics, Fujian Provincial Hospital, Fuzhou, 350001, P. R. China; Fujian Key Laboratory of Geriatrics, Fuzhou, 350001, P. R. China; Fujian Provincial Center for Geriatrics, Fuzhou, 350001, P. R. China; Shengli Clinical Medical College of Fujian Medical University, 134 East Street, Fuzhou, 350001, P. R. China; Fujian Provincial Institute of Clinical Geriatrics, Fujian Provincial Hospital134 East Street, Fuzhou, 350001, P. R. China; Fujian Key Laboratory of Geriatrics, 134 East Street, Fuzhou, 350001, P. R. China; Fujian Provincial Center for Geriatrics, 134 East Street, Fuzhou, 350001, P. R. China

**Keywords:** exercise, PGC-1α, DNA methylation, sarcopenia

## Abstract

Exercise training can significantly improve skeletal muscle mitochondrial function and has been proven to be highly relevant to alterations in skeletal muscle DNA methylation. However, it remains unclear whether late-in-life exercise has an effect on promoter methylation of PGC-1α, a key regulator of mitochondrial biogenesis. Here we employed two distinct exercise modalities, constant medium intensity exercise training (CMIT) and high-intensity interval exercise training (HIIT), to investigate their impacts on PGC-1α expression and methylation regulation in skeletal muscle of aged mice. The results revealed a notable decrease in PGC-1α expression in skeletal muscle of aged mice, accompanied by elevated methylation levels of the PGC-1α promoter, and increased DNA methyltransferase (DNMT) protein expressions. However, both forms of exercise training significantly corrected PGC-1α epigenetic changes, increased PGC-1α expression, and ameliorated skeletal muscle reduction. Furthermore, exercise training led to elevated expression of proteins related to mitochondrial biogenesis and energy metabolism in skeletal muscle, improving mitochondrial structure and function. In conclusion, late-in-life exercise improved skeletal muscle function, morphology, and mitochondria biogenesis, which may be associated with hypomethylation in promoters of PGC-1α and increased content of skeletal muscle PGC-1α. Notably, there was no clear difference between HIIT and CMIT in PGC-1α expression and skeletal muscle function.

## Graphical abstract

**Figure:**
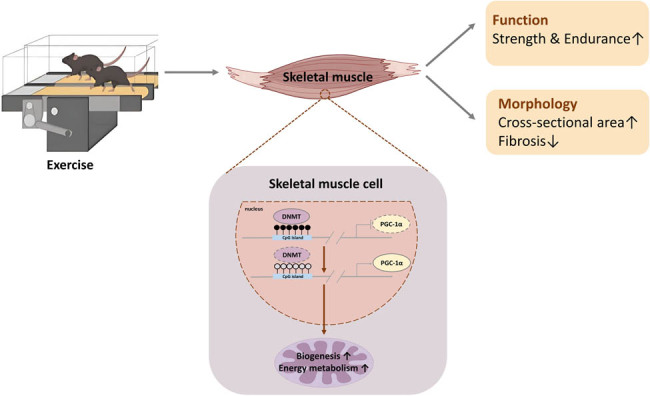

## 1 Introduction

The morphological changes and functional decline of skeletal muscle in old age have a great impact on life quality of the elderly [1]. The feasibility, safety, and effectiveness of long-term exercise training for the elderly to improve skeletal muscle function have been confirmed in clinical studies [2–4]. A small number of studies have suggested that high-intensity interval exercise training (HIIT) may be superior to the traditional constant medium intensity exercise training (CMIT), because it is a time-saving and effective strategy for improving exercise capacity [5,6].

Exercise training is intricately linked to changes in DNA methylation patterns and subsequent alterations in gene expression [7,8]. Recent studies indicate that HIIT, acute and chronic resistance exercise training, detraining, and retraining all induce modifications in methylome of human skeletal muscle [9,10]. Aging-related muscle loss is primarily attributed to mitochondrial dysfunction [11]. Peroxisome proliferator-activated receptor-γ coactivator-1α (PGC-1α) serves as a core regulator of mitochondrial energy metabolism and plays a fundamental role in maintaining a proper mitochondrial biogenesis, thereby playing a crucial role in ensuring normal energy metabolism in skeletal muscle [12]. Previous studies have suggested that exercise training or electrical stimulation may affect transcription levels by regulating the epigenetic modification of PGC-1α [13,14], while the effects of acute or short-term exercise on the promoter methylation of PGC-1α were still contradictory [14,15]. Furthermore, it remains unclear whether long-term exercise beginning in old age has an effect on promoter methylation of PGC-1α.

Therefore, this study aimed to explore the differences in DNA methylation level of PGC-1a promoter between CMIT and HIIT in aged mice, as well as its effects on skeletal muscle and mitochondrial function. Our findings will help to understand the underlying mechanisms by which exercise training affects skeletal muscle in old age and provide possible directions for the development of alternative therapies.

## 2 Materials and methods

### 2.1 Experimental animals

Healthy male C57BL/6 mice, weighing 30–40 g, were obtained from SPF Shanghai Slaughter Laboratory Animal Co. C57BL/6 mice were accommodated in the Experimental Animal Center of Fujian Medical University under consistent conditions of temperature (25°C), humidity (55 ± 5%), and 12 h artificial light and dark cycles. Unrestricted access to food and water was provided for 24 h. The mice were nourished and hydrated by the Experimental Animal Center of Fujian Medical University. The animal experiments were planned and executed in accordance with the relevant regulations of Fujian Medical University and were examined by the Experimental Animal Ethics Committee of Fujian Medical University (No. 2021-0363).


**Ethical approval:** The research related to animal use has been complied with all the relevant national regulations and institutional policies for the care and use of animals, and has been approved by the Experimental Animal Ethics Committee of Fujian Medical University (No. 2021-0363).

### 2.2 Grouping and exercise protocol

In this experiment, a total of 120 male C57BL/6 mice were used, with 30 being 3-month-old SPF-grade healthy males and 90 being 18-month-old SPF-grade healthy males. These mice were divided into four groups, each consisting of 30 mice (*n* = 30). The groups were named as follows: young group (Young group): normal feeding, 60 min per day on a stationary animal running platform; elderly stationary control group (Con group): normal feeding, 60 min per day on a stationary animal running platform; elderly continuous moderate intensity exercise training group (CMIT group): based on normal feeding, 8 weeks of a moderate intensity running platform training, each time the speed was set to 12 m/min and each training time was 46 min; and HIIT group: on the basis of normal feeding, 8 weeks of HIIT, at a speed of 17 m/min for 4 min and followed by intervals at a speed of 8 m/min for 3 min, repeated for six cycles, with a total distance of exercise which was equal to that of the CMIT group. All of the above lasted for 8 weeks.

### 2.3 Rotarod and grip strength tests

Mice were tested before and after the experiment for rotarod and forelimb grip strength. The animals were placed on the Panlab rotarod LE8505, and the trial started with the spindle rotating at 5 revolutions per minute (rpm) and gradually increased to 40 rpm over a period of 5 min. The time when the animal fell off the rotarod was recorded as the score. Each animal was tested three times, and the average score was used for analysis. The BIOSEB-BIO-GS3 grip strength meter measures the maximum forelimb grip strength in mice. The subjects were lifted from their cage by their tail base and hung above the grid until they firmly held onto the grid with their forepaws. The grid was slowly moved horizontally away from the mouse’s grip by pulling its tail. The maximal force was recorded. Every animal underwent six tests, with a 2 min break between each one. The average of the five tests was utilized for analysis.

### 2.4 Histological and immunohistochemical (IHC) staining

Fresh skeletal muscle tissue sections were stained with HE, Masson, and IHC. For IHC staining, the presence of brownish-yellow granules in the interstitium of skeletal muscle cells was observed under light microscopy as positive expression. The expression of PGC-1α in skeletal muscle tissues was assessed by measuring the area of positively stained area out of the total area using Image J software. The average percentage of the positively stained area (positively stained area/total area) was then calculated.

### 2.5 Average cross-sectional area (CSA) of gastrocnemius muscle fibers

Fresh skeletal muscle tissue was preserved with 4% paraformaldehyde and sliced into 5 µm sections. The tissue sections were deparaffinized, rehydrated, and HE stained to examine the morphological changes and damage to the gastrocnemius muscle. The images were observed using a light microscope with a 400× magnification. Image J software was utilized to determine the total area (µm^2^) of the skeletal muscle fibers in the gastrocnemius muscle. The average CSA of gastrocnemius fibers was determined by dividing the total muscle fiber area (µm^2^) by the number of muscle fibers.

### 2.6 Transmission electron microscopy

Samples of gastrocnemius muscle with a volume of 1 mm^3^ were obtained, fixed, washed, cut to 60–80 nm, and stained. Under an electron microscope, the ultrastructure of the mouse gastrocnemius muscle was examined.

### 2.7 Reverse transcription polymerase chain reaction (RT-PCR)

Mouse skeletal muscle total RNA was isolated and reverse transcribed into cDNA for RT-PCR analysis. RT-PCR was carried out using the 2^−ΔΔCt^ method to measure gene expression. The primer sequences were as follows:

PGC-1α-F: CGCTGCTCTTGAGAATGGATAT;

PGC-1α-R: GTCATACTTGCTCTCTTGGTGGAA;

ACTB-F: TGTCCACCTTCCAGCAGATGT;

ACTB-R: AGCTCAGTAACAGTCCGCCTAG.

### 2.8 Western blot

Western blot was performed on mouse gastrocnemius muscle samples. Primary antibodies used included PGC-1α, DNA methyltransferase (DNMT1), DNMT3A, DNMT3B, β-Tubulin, ERR, NRF1, CPT1B, GLUT4 (Abclonal, Wuhan, China), TFAM (Proteintech, Wuhan, China), and AMPK (Immunoway, Suzhou, China). Exposure was evaluated using a chemiluminescence imager and the grayscale values of each protein band were quantitatively analyzed using ImageJ software; the relative ratios were calculated by the optical density values of the bands of the target proteins in each group against the optical density values of the internal reference proteins.

### 2.9 Methylation-specific PCR (MSP)

The CpG islands in the mouse PGC-1α promoter region were retrieved using the Online MetPrimer software (http://www.urogene.org/methprimer/). Mouse skeletal muscle genomic DNA was extracted and used for bisulfite conversion in the MSP assay. The MSP primers designed by Methprimer were as follows:

methylated-F: TGGAATGGTTGAGAAGGTAGTTATC;

methylated-R: ACGTCTATTTAAAAAACTCACCGAA;

unmethylated-F: GGAATGGTTGAGAAGGTAGTTATTG;

unmethylated-R: ACATCTATTTAAAAAACTCACCAAA.

Afterward, the MSP product were examined on a 2% agarose gel, exposed for development on the chemiluminescence imager, and densitometric analysis was performed using ImageJ software.

### 2.10 Mitochondrial DNA (mt DNA) content assay

Mouse skeletal muscle genomic DNA was extracted for mt DNA assay. The copy number of mt DNA was quantified by measuring NADH dehydrogenase subunit 1(ND1) and normalized to the nuclear DNA lipoprotein lipase (LPL) gene using RT-PCR. The primer sequences were as follows:

ND1-F: CACTATTCGGAGCTTTACG;

ND1-R: TGTTCTGCTAGGGTTGA;

LPL-F: GAAAGGGCTGCCTGAGTT;

LPL-R: TAGGGCATCTGAGAGAGCGAGT.

### 2.11 Statistical analysis

The following results were analyzed for the surviving mice and calculated using SPSS24.0 statistical software. The mean ± standard deviation was used for the measurement data conforming to normal distribution and the paired *t*-test was used for the before–after comparison between the same groups, the group *t*-test was used for the comparison between two groups and the one-way ANOVA was used for the comparison between multiple groups. *p* < 0.05 was considered statistically significant.

## 3 Results

### 3.1 Late-in-life exercise enhances skeletal muscle function and improves its morphology

**Figure 1:**
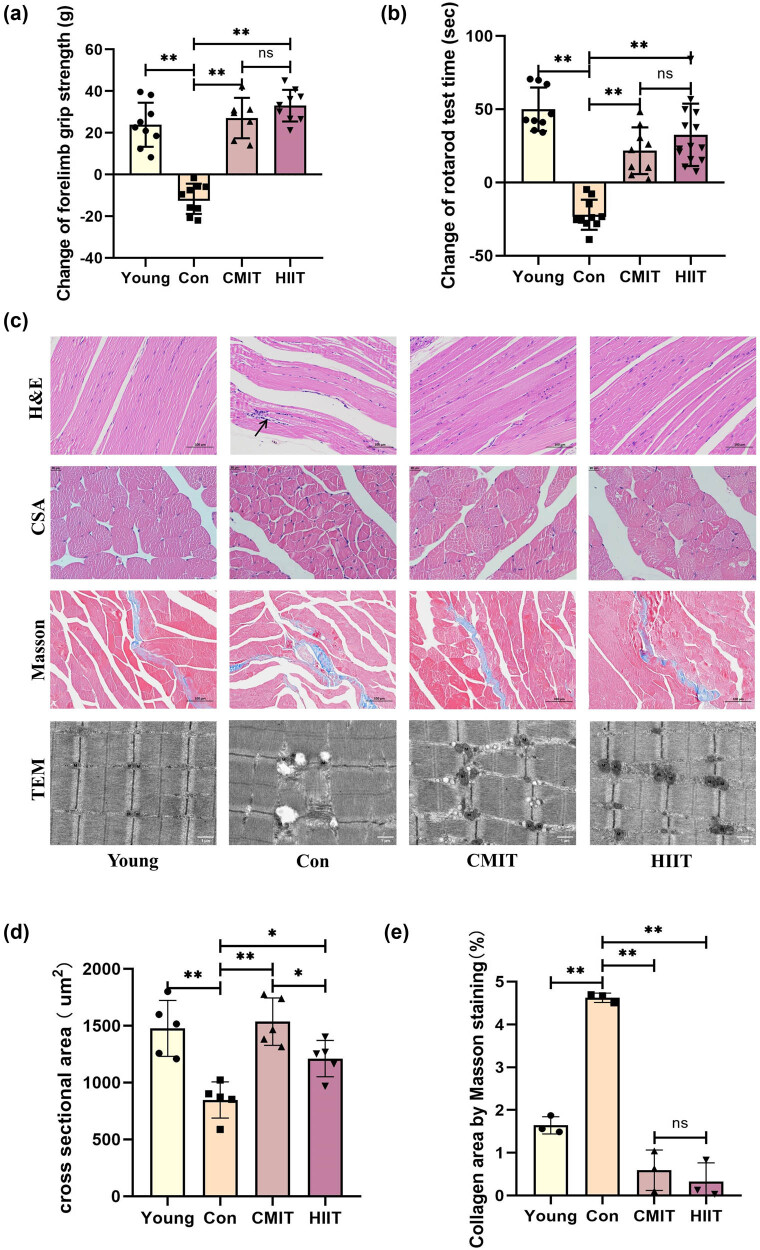
Late-in-life exercise enhances skeletal muscle function and morphology. (a) Change of forelimb grip strength after intervention. (b) Change of the Rotarod test time after intervention. (c) Representative images of H&E, average CSA, Masson staining, and TEM analysis of mouse gastrocnemius muscle tissue. Scale bar = 100 μm for H&E and Masson; scale bar = 20 μm for CSA; scale bar = 1 μm for TEM. (d) Relative quantification of average CSA of mouse gastrocnemius muscle. (e) Relative quantification of the degree of fibrosis of mouse skeletal muscle tissue (collagen fiber staining area/total area). *P < 0.05, **P < 0.01, ns represents no significant difference. CSA: cross-sectional area; TEM: transmission electron microscopy.

The test for forelimb grip strength directly reflects the muscular strength of mice, while the rotarod test assesses their motor coordination and fatigue endurance. After 8 weeks, mice in the Con group showed a decline in both maximum grip strength and rotarod performance, while those in the CMIT and HIIT groups exhibited increases in both measures. Interestingly, mice in the HIIT group displayed no significant difference compared to the CMIT group in terms of grip strength and rotarod performance (Figure 1a and b).

HE staining showed minor myocyte lysis and fragmentation at the periphery of skeletal muscle tissues in the Con group, accompanied by a slight infiltration of inflammatory cells in the interstitium (black arrowhead). Both the CMIT and HIIT groups exhibited marked improvement in skeletal muscle organization, myocyte lysis, and inflammation infiltration (Figure 1c). The CSA of gastrocnemius muscle fibers is a critical determinant of muscle strength [16]. Compared to the Young group, mice in the Con group exhibited a decrease in the average CSA of gastrocnemius fibers. However, both exercise modes significantly increased the average CSA of gastrocnemius fibers compared to the Con group, with the CMIT group showing a notably higher average CSA than the HIIT group (Figure 1c and d). Masson staining revealed that fibrosis in the skeletal muscle of the aged mice was increased markedly relative to that of its younger counterparts. However, both the CMIT and HIIT groups showed a significant reduction in skeletal muscle fibrosis compared to the Con group. Although the degree of fibrosis was lower in the HIIT group than in the CMIT group, the disparity did not reach statistical significance (Figure 1c and e). The transmission electron microscopy (TEM) analysis of Con group showed swollen mitochondria with disrupted crista and decreased electron density, as well as increased lipid droplets around mitochondria. In contrast, the mitochondrial structure of CMIT and HIIT groups displayed significantly improved mitochondrial structure, with noticeable mitochondrial aggregation compared to the Con group (Figure 1c).

### 3.2 Impact of late-in-life exercise on expression of PGC-1a via DNA methylation regulation

**Figure 2:**
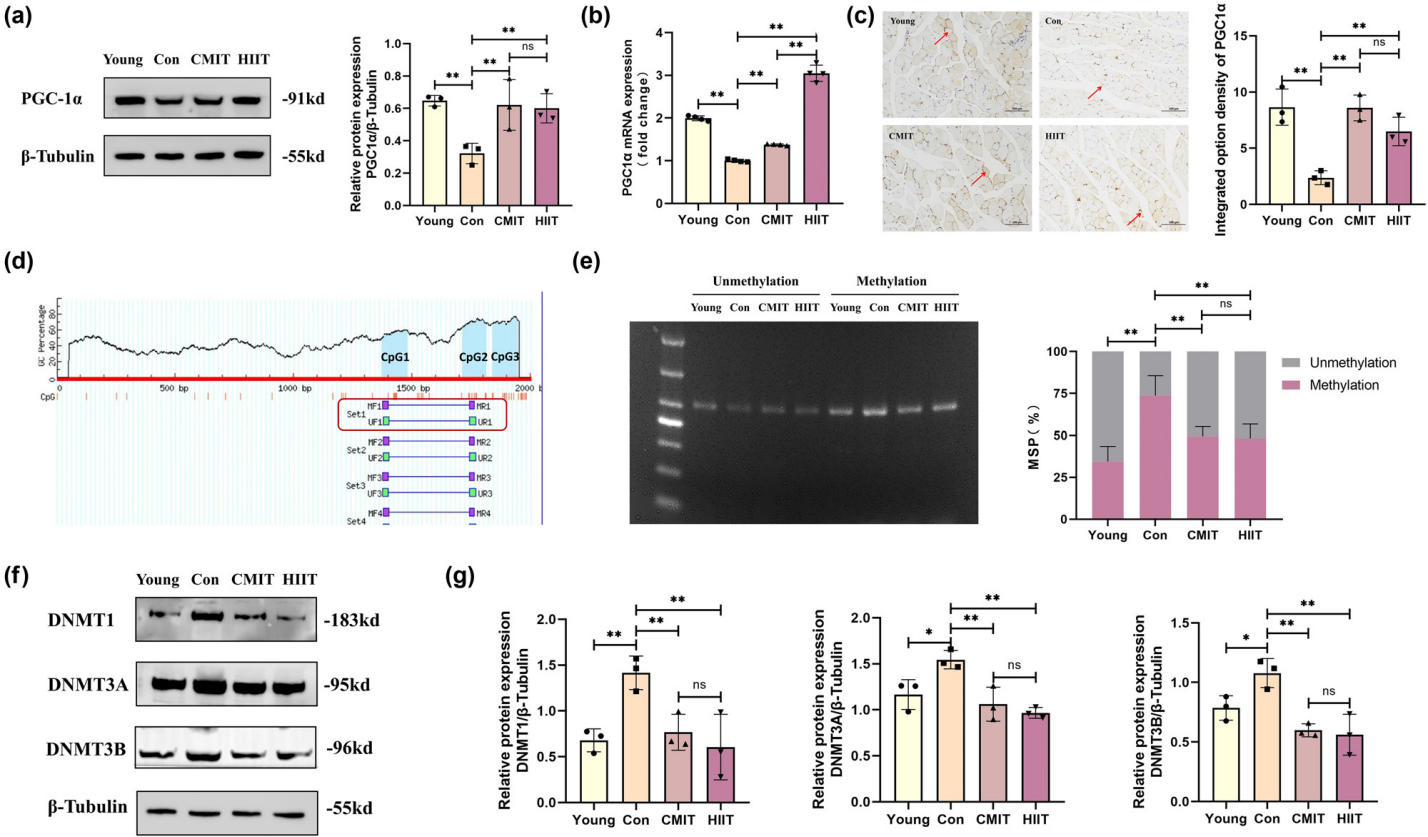
Impact of late-in-life exercise on expression of PGC-1α via DNA methylation regulation. (a) Western blot for protein levels of PGC-1α in mouse skeletal muscle. Representative images are shown on the left; quantitative data for the PGC-1α protein level are shown on the right. (b) PGC-1α mRNA relative expression. (c) IHC staining of PGC-1α in mouse skeletal muscle tissue. Representative images (red arrow is PGC-1α-positive staining area) are shown on the left; quantitative data are shown on the right. Scale bars = 100 μm. (d) Prediction of CpG islands of PGC-1α promoter (−2,000–0) in mouse skeletal muscle. (e) The MSP method was applied to examine the methylation status of PGC-1α promoter in mouse skeletal muscle. Representative agarose gel analyses of MSP products are shown on the left; quantitative data are shown on the right. (f) Western blot for protein levels of DNMT family (DNMT1, DNMT3A, and DNMT3B) in mouse skeletal muscle. (g) Relative quantitative expression of DNMT proteins. *P < 0.05, **P < 0.01, ns represents no significant difference. DNMT: DNA methyltransferase.

The mRNA and protein levels of PGC-1α in skeletal muscle of mice from the Con group were significantly lower than those in the Young group. Conversely, both the CMIT and HIIT groups exhibited a notable increase of the mRNA and protein levels of PGC-1α in the skeletal muscle compared to the Con group. Although the PGC-1α mRNA expression in the skeletal muscle of mice in the HIIT group surpassed that of the CMIT group significantly, there was no significant variance in PGC-1α protein expression between the two groups (Figure 2a and b). IHC analysis of the mouse skeletal muscle confirmed the heightened protein levels of PGC-1α in the CMIT and HIIT groups (Figure 2c).

CpG islands situated in the promoter region are prone to methylation alterations, which diminish transcription factor binding and consequently affect gene expression [17]. To investigate the reason for high PGC-1α expression in the CMIT and HIIT groups, we determined the presence of CpG islands within the PGC-1α promoter sequence using the MethPrimer software. Three CpG islands (CpG1-3) were found in the promoter region of the mouse PGC-1α gene, and the current MSP primers were designed targeting CpG1-2 (−1,377−1,835 bp) (Figure 2d). The MSP method was applied to assess the methylation status of specific position in the PGC-1α promoter. The results revealed an elevation in PGC-1α promoter methylation in skeletal muscle tissues of mice in the Con group relative to the Young group. The methylation level of the PGC-1α promoter in skeletal muscle tissues of mice in both CMIT and HIIT groups exhibited a noteworthy decline compared to the Con group, with no statistically significant disparity between the CMIT and HIIT groups (Figure 2e).

Given that DNA methylation is mediated by its key enzyme DNMT, we subsequently detected the protein expressions of DNMT family, including DNMT1, DNMT3A, and DNMT3B in skeletal muscle tissues. The research observed increased expression of DNMT1, DNMT3A, and DNMT3B proteins in the skeletal muscle tissues of mice in the Con group in comparison to the Young group. However, these protein alterations were reversed in both groups of aged mice following different exercise training interventions, and there were no statistically significant differences in the expression levels of DNMT proteins between the CMIT and HIIT groups (Figure 2f and g).

### 3.3 Impact of late-in-life exercise on mitochondrial biogenesis and energy metabolism

**Figure 3:**
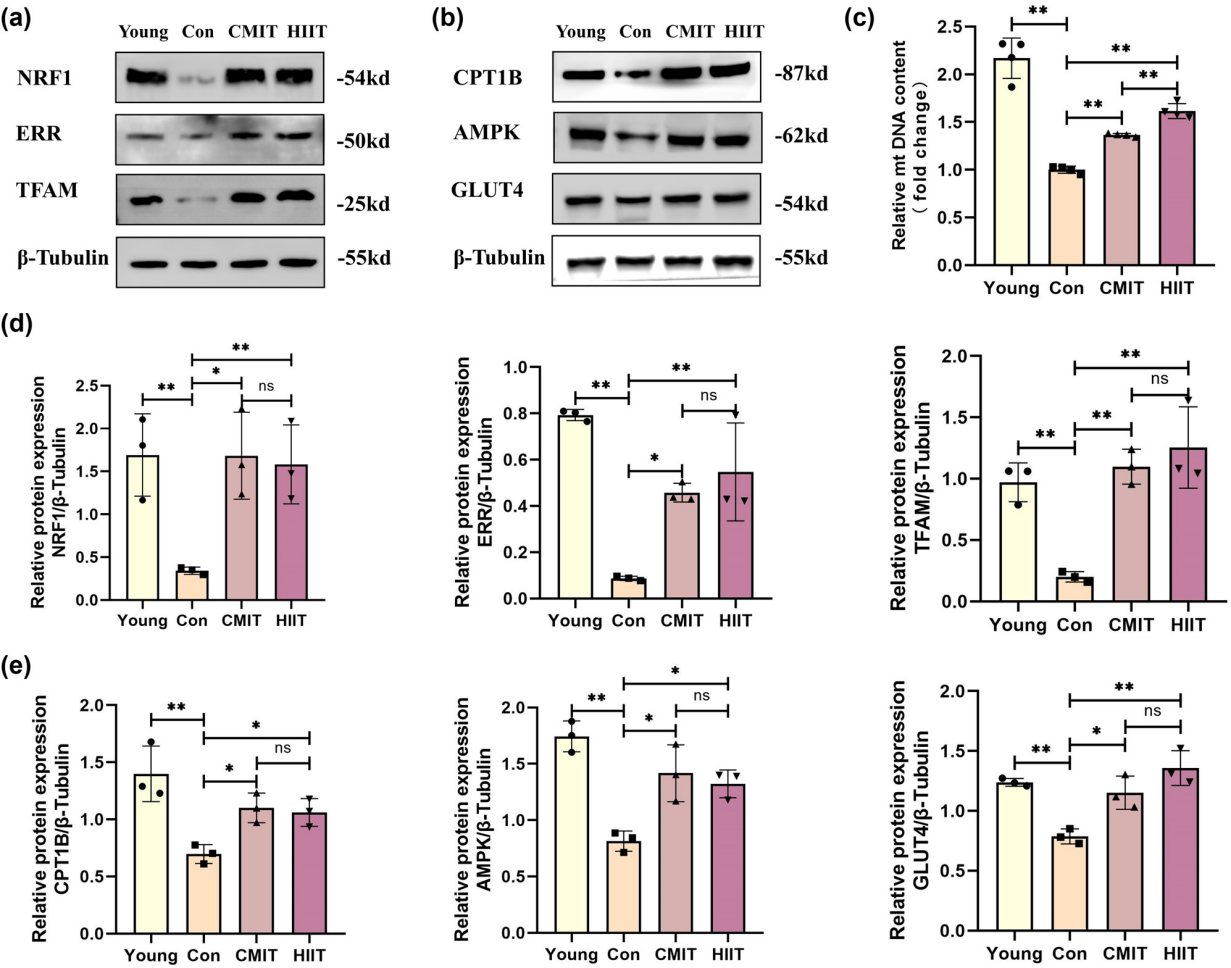
Impact of late-in-life exercise on mitochondrial biogenesis and energy metabolism. (a) Western blot for protein levels of NRF1, TFAM, and ERR in mouse skeletal muscle. (b) Western blot for protein levels of AMPK, GLUT4, and CPT1B in mouse skeletal muscle. (c) mt DNA content of mouse skeletal muscle. (d) Relative quantification of mitochondrial biogenesis-related proteins. (e) Relative quantification of energy metabolism-related proteins. *P < 0.05, **P < 0.01, ns represents no significant difference.

To further investigate the impact of increased PGC-1α expression on mitochondrial function during late-in-life exercise, the research measured the expression levels of protein related to mitochondrial biogenesis (NRF1, TFAM, and ERR) and energy metabolism (AMPK, GLUT4, and CPT1B) in skeletal muscle tissues of mice. It was found that the protein levels of NRF1, TFAM, ERR, AMPK, GLUT4, and CPT1B in skeletal muscle tissues of mice in the Con group were lower in comparison to those in the Young group. Conversely, CMIT and HIIT groups showed elevated expression levels of these proteins compared to the Con group. The levels of mitochondrial biogenesis and energy metabolism-related proteins were not statistically different between the CMIT and HIIT groups (Figure 3a, b, d, and e). Subsequently, mt DNA was detected. The skeletal muscle mt DNA content of mice in the Con group decreased compared to the Young group; conversely, it increased in both the CMIT and HIIT groups compared to the Con group. Remarkably, the skeletal muscle mt DNA content of mice in the HIIT group surpassed that of the CMIT group significantly (Figure 3c).

## 4 Discussion

In this research, we observed that late-in-life exercise can ameliorate aging-related morphological changes in skeletal muscle and enhance skeletal muscle function, which may be associated with its effect on PGC-1α expression via PGC-1α promoter methylation regulation. In addition, exercise-training intervention reversed the mitochondria structural abnormalities of skeletal muscle in aged mice and significantly increased the expression levels of proteins related to mitochondrial biogenesis and energy metabolism. There seemed no clear difference between HIIT and CMIT in PGC-1α expression and skeletal muscle function.

PGC-1α has attracted considerable attention as a pivotal regulator of mitochondrial function, playing a central role in maintaining normal energy metabolism and mitochondria homeostasis in skeletal muscle. Previous studies have shown age-related decreases in PGC-1α protein content along with decreased numbers and impaired function of mitochondria in skeletal muscle in rats, mice, and humans [18–20], which were also observed in our study.

Barrès et al. [21] provided a systematic study about methylation of PGC-1α in skeletal muscle from patients with diabetes. They used whole-genome promoter methylation to identify hypermethylation of PGC-1α. They also reported DNMT3B rather than DNMT1 or DNMT3A related to fatty-acid-induced methylation of PGC-1α promoter. Our results were generally consistent with previous findings in human being that both acute [15] and chronic [22] exercise altered the promoter methylation of PGC-1α. When exploring the potential cause, we found a global decrease of DNMT family, which was different with Romain’s finding. However, the current result was partly in line with other studies where exercise has been shown to repress DNMT expression levels in human plasma and in human and mouse femurs [23–26]. Although our finding led to a hypothesis that exercise training could ameliorate aging-related muscle loss by reducing PGC-1α promoter methylation and enhancing PGC-1α expression, the expression of DNMTs were still far from to conclude the causality of methylation. And we also had to consider enzymatic activity and other regulators’ expression, such as ten-eleven translocation enzymes, when interpreting the mechanism.

Numerous studies have demonstrated that PGC-1α in skeletal muscle enhances skeletal muscle mitochondrial biogenesis and energy metabolism as well as improves mitochondrial structure and function [27–30]. In this study, we detected the expression levels of proteins related to these processes, alongside assessing mitochondrial structure in all groups of mice. The current findings suggested that late-in-life exercise upregulated expression of protein crucial for mitochondrial biogenesis and energy metabolism, improved mitochondrial ultrastructure and increased mt DNA content in aged mouse skeletal muscle, potentially linked to increased PGC-1α expression. This is consistent with existing study results [18,31], indicating that exercise-induced improvement of skeletal muscle mitochondria in aged mice is partially reliant on increased content of skeletal muscle PGC-1α.

Herein, we employed two common exercise modalities, CMIT and HIIT, to investigate their effects on PGC-1α expression and methylation levels in skeletal muscle of aged mice. Although some studies have indicated the potential superiority of HIIT over CMIT in improving skeletal muscle mitochondrial quantity, mitochondrial adaptability, and oxidative capacity [32–34], our study merely observed significant differences in skeletal muscle average CSA and mt DNA content between CMIT and HIIT. Moreover, we found no notable distinctions between the two exercise modalities in terms of PGC-1α expression, mitochondria mobilization, and improvement of skeletal muscle morphology and function in old age. Thus, currently we did not recommend specific exercise type over another in older adults.

The present study has several limitations. It was a preliminary exploration of the role of PGC-1α methylation in mitigating muscle loss in aged mice through exercise, yet it does not directly elucidate the causal relationship and exact mechanism of altered PGC-1α methylation on exercise-driven skeletal muscular alterations in old age. In addition, the study did not explore the possible effects of other epigenetic modifications on PGC-1α transcription, such as histone modification and non-coding RNA, nor did it explore the effects of exercise on fat content, or on other organ function. Therefore, it cannot be concluded that HIIT and CMIT have similar effects in all aspects in old age.

## 5 Conclusion

Late-in-life exercise improves skeletal muscle function and morphology, reverses the mitochondria structural abnormalities of skeletal muscle in aged mice, and affects the expression levels of proteins related to mitochondrial biogenesis and energy metabolism, which may be linked to its effect on hypomethylation in promoters of PGC-1a and elevated protein and mRNA expression of PGC-1α. Notably, there was no clear difference between HIIT and CMIT in PGC-1α expression and skeletal muscle function.
